# Supplementary material for: Enzymatic incorporation of an isotope-labeled adenine into RNA for the study of conformational dynamics by NMR
Source: PLoS One. 2022 Jul 8;17(7):e0264662. doi: 10.1371/journal.pone.0264662 (PMC9269771; doi:10.1371/journal.pone.0264662)

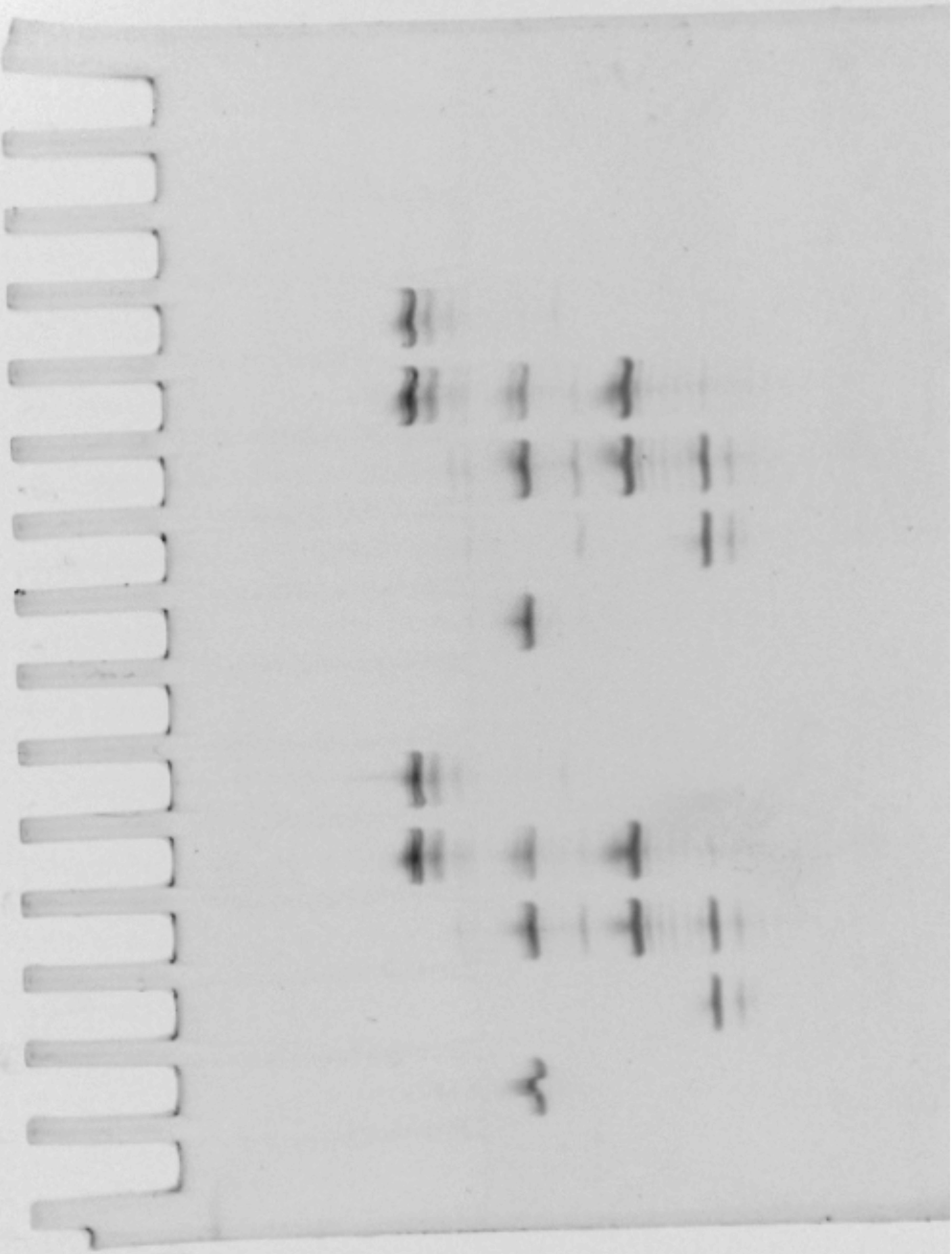

Supplement: S1 Raw image — (PDF) [file pone.0264662.s001.pdf]
